# Lessons On Using Health Research & Technology To Broaden Resources For Older Adults Across The State

**DOI:** 10.1093/geroni/igab046.366

**Published:** 2021-12-17

**Authors:** Alexis Travis

**Affiliations:** Michigan Department of Health and Human Services, Lansing, Michigan, United States

## Abstract

The vision of the Michigan state unit on aging is for residents to live well and thrive as they age. The COVID-19 pandemic exacerbated the existing problem of older adult social isolation. Social engagement and community involvement are keys to healthy aging. Combining state resources with the GetSetUp virtual community allowed for statewide connections and extended resources, creating an almost around-the-clock virtual senior center. Through customized courses the state was able to offer vaccine navigation sign-up classes, among other classes, to help older adults interact with essential health and aging services. As Michigan continues to work to address health equity and social determinants of health beyond the pandemic, technology designed specifically for older adults is an important component of programmatic offerings. It also allows for a public-private partnership opportunity to support older adults as they age.

